# Infrared Thermography to Assess Thermoregulatory Reactions of Female Buffaloes in a Humid Tropical Environment

**DOI:** 10.3389/fvets.2020.00180

**Published:** 2020-05-19

**Authors:** Carolina Carvalho Brcko, Jamile Andréa Rodrigues da Silva, Lucieta Guerreiro Martorano, Reíssa Alves Vilela, Benjamim de Souza Nahúm, André Guimarães Maciele Silva, Antônio Vinícius Corrêa Barbosa, Andréia Santana Bezerra, José de Brito Lourenço Júnior

**Affiliations:** ^1^Institute of Veterinary Medicine, Federal University of Pará, Castanhal, Brazil; ^2^Institute of Animal Health and Production, Federal Rural University of the Amazon, Belém, Brazil; ^3^Embrapa Eastern Amazon, Belém, Brazil

**Keywords:** buffaloes, climate, heat, images, thermoregulation

## Abstract

This study employed infrared thermography to assess the thermoregulatory responses of female buffaloes reared in a hot and humid climate as a function of variations in time and the thermal environment, and to correlate rectal temperature with that of different body areas. The trial was carried out at Embrapa Eastern Amazon (01°26'S and 48°24'W) in Belém, PA, Brazil. Twenty-four female buffaloes fed exclusively on pasture and exposed to the sun throughout the day in an open area were used. The animals were taken back to the corral at 6 A.M., 9 A.M., 12 P.M., 3 P.M., 6 P.M., and 9 P.M. for measurements of rectal temperature (RT), respiratory rate (RR), and body surface temperature (BST) using infrared thermography (IRT). Data on air temperature (AT), relative air humidity (RH), wind velocity (WV), and solar radiation (SR) were also recorded. A quadratic effect of AT, RH, SR, RR, and RT (*P* < 0.01) was found as a function of measurement time. Between 12 P.M. and 3 P.M., AT, SR, RR, and RT values were the highest, while RH values were the lowest (*P* < 0.05). WV was the highest between 12 P.M. and 9 P.M. A difference was found in RR and RT among the day's times, with the highest values at 12 P.M. and 3 P.M. (P <0.05). The IRTs have a quadratic effect as a function of day's times, with the highest levels between 12 P.M. and 6 P.M. and a reduction at 9 P.M. All IRTs are correlated with the physiological variables RR and RT, except for the IRT of the side of the back, which is not correlated with RT. The IRTs of the eye and cheek are correlated with RT (*P* < 0.01) and with RR (*P* < 0.05). IRT was efficient in evaluating the thermal stress of female buffaloes in hot and humid climates, and the technique can be used to evaluate the body temperature of these animals, since the RT was positively correlated with the temperature of the eye and cheek.

## Introduction

Buffaloes (*Bubalus bubalis*) were introduced in Brazil in 1895, in the Island of Marajó, PA (1), and the current herd is estimated at 1.4 million heads. The country's North region accounts for most of that with 922,600 heads, or 66.4% of the national herd, followed by the Southeast and Northeast regions, with 188,100 and 125,300 thousand heads, respectively. Currently, buffalo farming in this region provides meat, milk, leather, and horns and is concentrated mainly on small farms (2).

Buffaloes are known for their rusticity in adverse environments and represent an option to make use of areas in a ranch to which bovines do not adapt (3). However, the difficulty these animals have in releasing body heat under high temperature and humidity may negatively impact their productive and reproductive performances (4). Buffaloes in hot conditions have increased blood flow on their skin surfaces so as to raise its temperature and facilitate heat loss through conduction when the animals are immersed in mud or water (5).

The use of infrared thermography (IRT) poses an alternative to assess the body surface temperature (BST) since it enables obtaining the heat irradiating in the infrared spectrum without interfering on animals' behavior (6). This technique is innovative as it can be used with no direct interaction with the animal, thus being considered a non-invasive tool.

In face of that, the present research aimed to assess the thermoregulatory responses of female buffaloes reared in the hot and humid climate of the Eastern Amazon as a function of variations in time and thermal environment, besides employing thermography to map the BST of buffaloes in order to find the area that is most associated with rectal temperature (RT), hence contributing to animal biometeorology studies.

## Materials and Methods

The experiment was approved by the Ethics Committee on Experimental Animal Research of the Federal University of Pará under the protocol BIO 120-13. The experimental sample consisted of 24 non-pregnant, non-lactating, clinically healthy female crossbred Murrah/Mediterranean buffaloes (*Bubalus bubalis bubalis*) whose initial mean age and weight were 54 ± 7 months and 503.1 ± 23 kg, respectively. The animals were selected according to their body score aiming at greater uniformity and were kept in 1-ha pens with *Brachiaria brizantha* (cv. Marandu) grass in a rotational system with no access to shade or ponds and with drinking water and mineral salt *ad libitum*.

The trial lasted for six consecutive days. The buffaloes were weighed before and after the trial. Such information was used to calculate heat storage and later to calculate the accumulated heat following the recommendations by McGovern and Bruce (7).

Heat storage calculation, in kelvin kg^−1^ h^−1^:

$$\begin{array}{l} \Delta RT=\frac{\left(3,600.Harm.A\right)\;}{\left(Mv.Hb\right)\;}\text{ where}: \end{array}$$

ΔRT—difference in RT;

H_arm_–stored heat (W.m^−2^);

A—animal's surface (m^2^) calculated by the equation: A=0.13.${\text{M}}_{\text{v}}^{0.556}$;

M_v_–body mass (kg);

H_b_–animal's specific heat (3,400 kJ kg^−1^ K^−1^).

About 30 min prior to collections, animals were taken to the corral about 300 m away from the pen, where they were contained at 6 A.M., 9 A.M., 12 P.M., 3 P.M., 6 P.M., and 9 P.M. for the measurement of the physiological variables of RT, respiratory rate (RR), and BST measured through IRT, with ± 1°C accuracy and a spectral range of 7.5–13.0 μm.

A clinical veterinary thermometer graded in Celsius, with decimal accuracy, was inserted 5 cm into animals' rectum for 1 min to measure the RT. RR was obtained through inspection and by counting the thoracic-abdominal movements for 1 min with the aid of a stopwatch. The animals randomly entered the holding pens.

**Figure 1:**
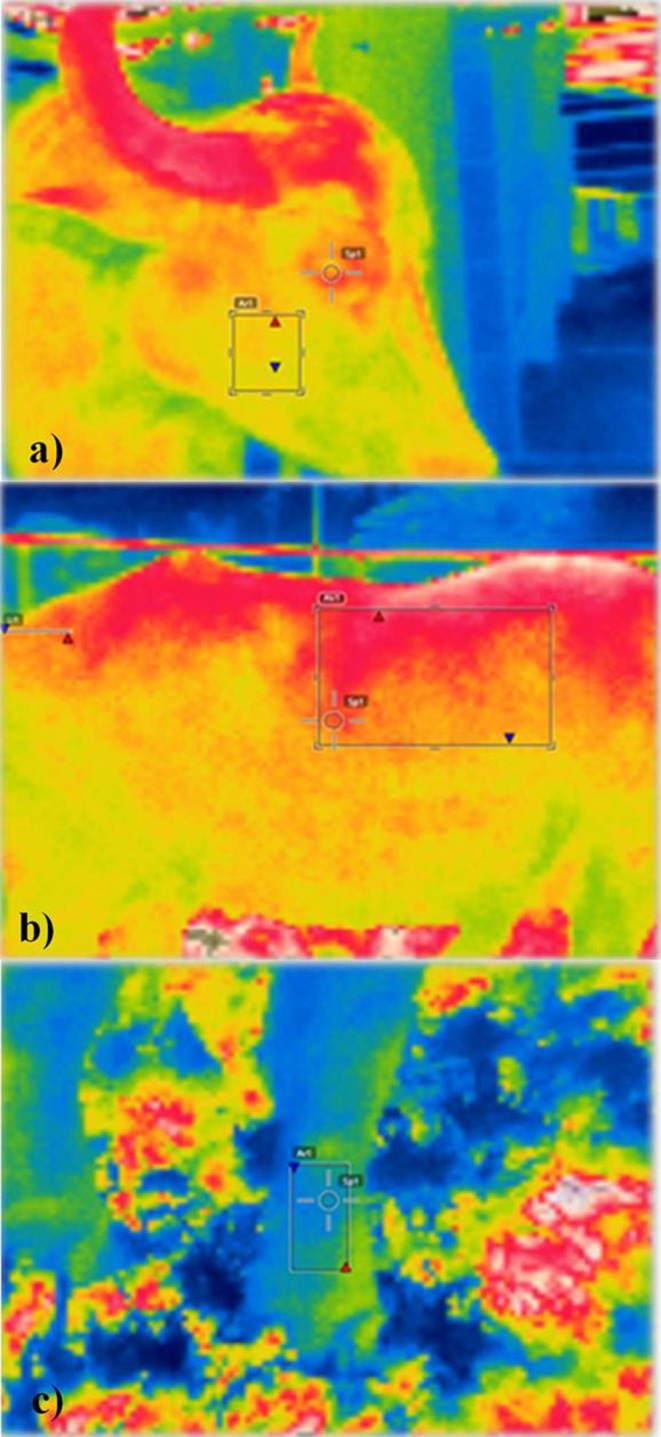
**(a)** Cheek, eye, **(b)** side of the back, tail insertion point, and **(c)** ergot of female buffaloes reared in the Eastern Amazon.

The IRTs were obtained using a FLIR T E30 thermographic camera with automatic calibration. The measurements were determined by the average readings of two areas delimited in the image by markers. All images were acquired from the animals' right side so as to reflect the actual IRT fluctuation, thus preventing digestive processes in the rumen from impacting the increase in BST. Each thermogram was saved to a memory card and later analyzed in the software FLIR Tools, where the mean temperatures of each region were obtained considering emissivity of 0.98. Images were taken from five areas, namely, side of the back, tail insertion, eye, cheek, and hind ergot (Figure 1). Approximately 2,592 thermographic images were acquired during collection days. The ones that lacked the clarity required for the analysis in the software were excluded.

The distance used to take the photographs (distance between the camera and the evaluated area) was 30 cm for eye, cheek, and hind ergot and 1 m for side of the back and tail insertion.

The meteorological data of air temperature (AT,°C) and relative air humidity (RH, %) were recorded using a U12-012 HOBO® data logger (Onset, Brazil) installed at the experimental site. A TAD-800 portable digital thermo-anemometer (Instrutherm, São Paulo, Brazil) was used to measure wind velocity (WV, m/s). Solar radiation (SR, MJ.m^−2^) was obtained from INMET's (National Institute of Meteorology) station 3.5 km away from the experimental site. The environmental variables were read along the day at the same time as the physiological variables.

The statistical analysis of data was performed in the software Statistical Analysis System (SAS 9.1). The physiological responses (RT, RR, BST, and IRT) and climate variables (AT, RH, WV, and SR) were analyzed using general linear models (GLMs) with two statistical models. In the first one, for physiological variables, repeated measures are made on the same experimental unit (animal) over a period of time. The model is represented by: *Y*_*ijk*_ = μ + *a*_*i*_ + *b*_*icp*_ + *e*_*ijk*_, *y*_*ijk*_ = μ + α_*i*_ + *b*_*i*(*j*)_ + *e*_*ijk*_ in which *Y*_*ijk*_ refers to the value of the ith animal obtained at the jth hour of the kth day; μμ is the overall mean; *a*_*i*_ is the fixed effect of the jth hour of the day; *b*_*icp*_ is the random effect of the ith animal belonging to the jth hour of the day; and *e*_*ijk*_ refers to the residual including the random error. The second statistical model, for climate variables, is: *Y*_*jk*_ = μ + *aj* + *e*_*jk*_, in which *Y*_*jk*_ refers to the value obtained at the jth hour of the kth day; μ is the overall mean; *aj* is the fixed effect of the jth hour of the day; and *e*_*jk*_ refers to the residual including the random error.

To determine the linear relationship between BST [eye temperature (°C), cheek temperature (°C), side-of-back temperature (°C), tail temperature (°C), and ergot temperature (°C)] and RR and RT, Pearson correlation was calculated by the procedure PROC CORR in SAS. To perform regression in SAS between RR and time of the day and RT and time of the day, the procedure PROC REG (means adjusted by least square) was employed by a quadratic polynomial model: *Y* = β_0_ + β_1_*X* + β_2_*X*^2^, $Y=\beta_{0}+\beta_{1}X+\beta_{2}X^{2}$in which *Y* is the physiological variable [eye temperature (°C), cheek temperature (°C), side-of-back temperature (°C), tail temperature (°C), ergot temperature (°C), RR (mov/min), and RT (°C)] and *X* is the time of the day (6 A.M., 9 A.M., 12 P.M., 3 P.M., and 9 P.M.).

## Results

An interaction can be seen among the meteorological variables and the day's times. The mean, maximum, and minimum values, the number of observations (N), standard deviation (SD), and coefficient of variation (CV) of climate variables and RR and RT of female buffaloes are presented in Table 1.

**Table 1:** Number of observations (N), mean, standard deviation (SD), coefficient of variation (CV), minimum (Min), and maximum (Max) values of agrometeorological variables and RR and RT of female buffaloes during the experimental period in the Eastern Amazon.

**Variable**	**N**	**Mean**	**SD**	**CV (%)**	**Min**	**Max**
AT	858	29.26	3.74	12.78	23.14	37.28
RH	858	74.50	10.97	14.72	47.62	90.06
THI	858	80.56	4.21	5.22	72.45	87.26
SR	858	968.34	1,013.52	102.76	−3.54	2,844
WV	858	0.97	0.81	83.50	0	2.5
RR	858	27.5	15.4	56.00	11	103
RT	858	38.8	0.48	1.24	30	40.7

*AT, air temperature (°C); RH, relative humidity (%); THI, temperature and humidity index; SR, solar radiation (kJ m^−2^); WV, wind velocity (m s^−1^)*.

The regression analysis associated with AT as a function of measurement time showed a quadratic model (*P* < 0.01). By deriving the equation, the highest AT value of 32.9°C was found at 1:42 P.M. It was observed that RH's effect is the opposite of AT's, with the lowest value of 64% at 1:27 P.M. (*P* < 0.05).

A quadratic effect was found as a function of assessment times in the regression associated with SR (*P* < 0.01). The maximum radiation value, by deriving the equation, was 1,639 MJ m^−2^ at 1:35 P.M.

**Figure 2:**
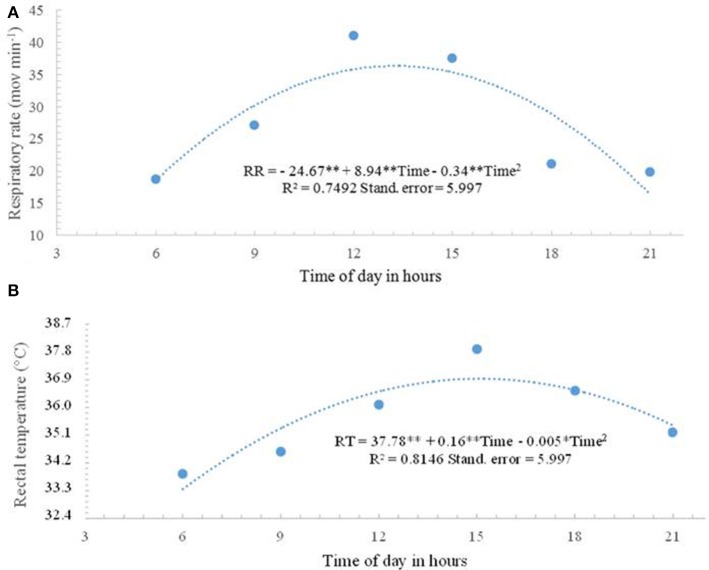
**(A)** Respiratory rate (RR) and **(B)** rectal temperature (RT) of female buffaloes observed at 6 A.M., 9 A.M., 12 P.M., 3 P.M., 6 P.M., and 9 P.M. during the experimental period in the Eastern Amazon. *p < 0.05 **p < 0.01.

The regression analysis regarding RR showed a quadratic effect (*P* < 0.01) as a function of the time of day (Figure 2). By deriving the equation, the time of 1:28 P.M. was found for maximum RR, which would reach 36.08 mov min^−1^. A gradual increase in RR was observed up until 1 P.M., starting at 18.69 mov min^−1^ at 6 A.M., climbing to 27.08 mov min^−1^ at 9 A.M., and reaching mean values of 41.04 mov min^−1^ at 12 P.M.

**Figure 3:**
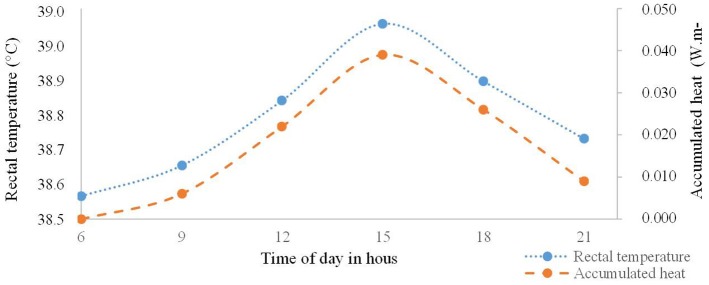
Thermal balance dynamics of female buffaloes observed at 6 A.M., 9 A.M., 12 P.M., 3 P.M., 6 P.M., and 9 P.M. during the experimental period in the Eastern Amazon.

**Figure 4:**
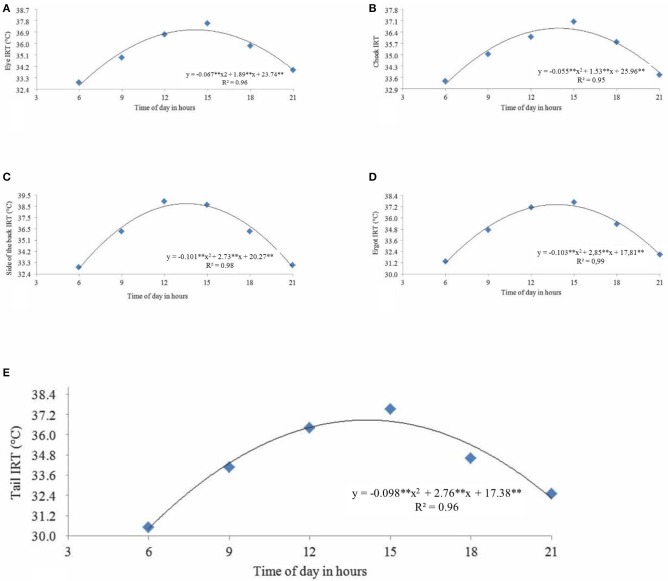
**(A)** Eye, **(B)** cheek, **(C)** side of the back, **(D)** ergot, and **(E)** tail infrared thermography (IRT) of female buffaloes observed at 6 A.M., 9 A.M., 12 P.M., 3 P.M., 6 P.M., and 9 P.M. during the experimental period in the Eastern Amazon. **p < 0.01.

A significant difference (*P* < 0.05) was found in RT along the day, with the highest value (39.01°C) at 3 P.M. The regression coefficient analysis associated with RT differed (*P* < 0.01) among the assessment times following a quadratic model, as seen in Figure 3. By deriving the equation, the time of 3:05 P.M. was obtained for maximum RT, which would reach 38.63°C. The thermal balance dynamics of female buffaloes observed at 6 A.M., 9 A.M., 12 P.M., 3 P.M., 6 P.M., and 9 P.M. during the experimental period in the Eastern Amazon is presented in Figure 4.

The regression analysis of IRTs showed a quadratic effect as a function of the times of the day. By deriving the equations, the following times would yield maximum IRT results for different parts of the body: 1:50 P.M. for the eye at 36.49°C (Figure 4A); 1:51 P.M. for the cheek at 36.6°C (Figure 4B); 1:51 P.M. for the back at 38.71°C (Figure 4C); 2:23 P.M. for the ergot at 37.01°C (Figure 4D); and 1:54 P.M. for the tail at 33.81°C (Figure 4E).

The correlation among IRTs and physiological variables is in Table 2. All IRTs are correlated with the RR and RT, except the IRT of the side of the back, which is not correlated with RT, although it is correlated (*P* < 0.01) with RR. The IRTs of the eye and cheek are correlated with RT (*P* < 0.01) and RR (*P* < 0.05). Likewise, the IRTs of the tail and ergot are correlated (*P* < 0.05) with RR and RT.

**Table 2:** Correlations among body surface temperatures assessed by infrared thermography and physiological variables of respiratory rate and rectal temperature of female buffaloes reared in a hot and humid climate.

**Body surface temperature**	**Respiratory rate**	**Rectal temperature**
Eye temperature (°C)	0.85303^*^	0.92904^**^
Cheek temperature (°C)	0.81573^*^	0.92226^**^
Side of back temperature (°C)	0.92965^**^	0.78412^NS^
Tail temperature (°C)	0.88633^*^	0.86125^*^
Ergot temperature (°C)	0.87408^*^	0.89005^*^

* *P < 0.05*;

** *P < 0.01*,

*^NS^, Nonsignificant*.

## Discussion

The highest AT value of 32.9°C was found at 1:42 P.M., which shows that the hotter climate at that time could be detrimental to the animals' thermal comfort. Under high AT and RH, animals dissipate heat and their physiological variable values change (8). It was observed that RH's effect is the opposite of AT's, with the lowest value of 64% at 1:27 P.M. (*P* < 0.05). Similar AT and RH values were reported by Silva et al. (9).

SR directly impacts the animals reared in a traditional system with no access to shade. Buffaloes are particularly susceptible to direct SR since they absorb a lot of heat because of their dark skin and sparse fur (10). The direct incidence of radiation onto the animals contributes to the increase in BST and further raises AT. Studies conducted in the Amazon region confirm that this environment is conducive to causing thermal stress in buffaloes, especially in the afternoon, and that the use of shading, as in a silvipastoral system, would facilitate body heat dissipation (11, 12).

The maximum radiation value by deriving the equation was 1,639 MJ m^−2^ at 1:35 P.M. Along the day, some moments are more or less favorable to animals' thermal comfort, and this process is mediated by the radiation balance, i.e., the difference between the radiation received and given back, which greatly varies along the day and the year and promotes daily and annual changes in AT (13).

The higher AT leads to a reduction in the thermal gradient between the animal and the environment, which hinders the sensible dissipation of accumulated metabolic heat (14). In this scenario, evaporative thermolysis becomes an important pathway to maintain this thermal balance. RR is an important indicator of animal thermal stress in hot environments because, in this situation, the animal's respiratory frequency increases to eliminate body heat through evaporative heat loss (15).

By deriving the equation, the time of 1:28 P.M. was found for maximum RR, which would reach 36.08 mov min^−1^. The higher RR values between 12 P.M. and 3 P.M. are associated with the increase in AT and SR. RR is relevant in the dissipation of endogenous heat in buffaloes.

According to Alam et al. (16), the thermal stimulation of peripheral thermoreceptors sets off evaporative thermolysis through polypnea. According to that reasoning, the animals accumulated heat along the day from SR, which led to higher RT despite them losing heat through the convection and latent pathways. After RT decreased, thermal balance was reestablished because the animals more effectively used sensible and latent thermolysis.

In the present research, a gradual increase in RR was observed up until 1 P.M., starting at 18.69 mov min^−1^ at 6 A.M., climbing to 27.08 mov min^−1^ at 9 A.M., and reaching mean values of 41.04 mov min^−1^ at 12 P.M. Similar values were reported by Silva et al. (17). The highest value of RT (39.01°C) was observed at 3 P.M. By deriving the equation, the time of 3:05 P.M. was obtained for maximum RT, which would reach 38.63°C.

It is seen that, even when AT was milder, RT values were beyond the normal range for buffaloes, i.e., 37.4 to 37.9°C (18). Since they have very pigmented skin and low fur density, when buffaloes are exposed to SR, the radiant energy is absorbed and transmitted following a centripetal heat flow (3). At 3 P.M., with SR of 2,443.1 MJ m^−2^, the animals' RT was very high (39.01°C), which suggests that the heat release mechanisms had become insufficient to maintain homeothermy. Later, as AT decreased, a change in heat flow following a centrifugal path was observed, with greater thermal inertia for heat dissipation. RT decreased between 6 P.M. and 9 P.M. to the point of reestablishing thermal balance.

Along the day, heat gain through radiation influenced the heat accumulated by the animals and, consequently, led to higher RT. The moment RT decreased, animals were able to regain thermal balance.

The following times would yield maximum IRT results for different parts of the body: 1:50 P.M. for the eye at 36.49°C; 1:51 P.M. for the cheek at 36.6°C; 1:51 P.M. for the back at 38.71°C; 2:23 P.M. for the ergot at 37.01°C; and 1:54 P.M. for the tail at 33.81°C. These results demonstrate that the hottest times of the day influence these variables.

The correlation among the IRTs and the physiological variables RR and RT may show the efficacy of these variables in indicating thermal stress. All IRTs are correlated with the RR and RT, except the IRT of the side of the back, which is not correlated with RT, although it is correlated (*P* < 0.01) with RR. The IRTs of the eye and cheek are correlated with RT (*P* < 0.01) and RR (*P* < 0.05). Likewise, the IRTs of the tail and ergot are correlated (*P* < 0.05) with RR and RT. These results show that the IRTs of the eye and cheek are the most appropriate to assess thermal stress in buffaloes since they have the best correlations with RT, which indicates thermal balance and is used to assess the adversity of the thermal environment (19).

Researches carried out on buffalo bulls (20) and dairy buffalos (21) also concluded that the IRT technique was efficient to identify the thermal stress in these animals raised in tropical climate.

Using the IRT technique, Barros et al. (20) observed, with this tool support, a correlation between the RT and the surface temperature of buffaloes in hot and humid climates. This technique has also supported the determination of semen quality through the correlation of scrotal surface temperature gradient with sperm quality parameters of buffalo bulls (22, 23).

## Conclusion

The heat dissipation mechanisms in buffaloes are efficient since, even when submitted to climate conditions that set off thermal stress, the animals were able to regain homeothermy as the environmental variables became milder.

The use of IRT is a good indicator of the BST of buffaloes and its association with thermoregulation. The IRTs of the eye and cheek were the most appropriate to determine thermal stress since they are best correlated with the animals' RT.

## Data Availability Statement

All datasets generated for this study are included in the article/supplementary material.

## Ethics Statement

The animal study was reviewed and approved by the Ethics Committee on Experimental Animal Research of the Federal University of Pará under the protocol BIO 120-13.

## Author Contributions

Experiment design: CB and JS. Experiment perform: CB, JS, LM, and RV. Data curation: CB, JS, and AS. Formal analysis: AS and AVCB. Writing-original draft: CB, JS, ASB, and JL. All authors edited and approved the final manuscript.

## Conflict of Interest

The authors declare that the research was conducted in the absence of any commercial or financial relationships that could be construed as a potential conflict of interest.
